# Lodenafil treatment in the monocrotaline model of pulmonary hypertension in rats*

**DOI:** 10.1590/S1806-37132014000400010

**Published:** 2014

**Authors:** Igor Bastos Polonio, Milena Marques Pagliareli Acencio, Rogério Pazetti, Francine Maria de Almeida, Bárbara Soares da Silva, Karina Aparecida Bonifácio Pereira, Rogério Souza

**Affiliations:** Department of Pulmonology, Santa Casa School of Medical Sciences in São Paulo, São Paulo, Brazil; Laboratory for Pleural Studies, Department of Pulmonology, University of São Paulo School of Medicine, São Paulo, Brazil; Laboratório de Investigação Médica 61 (LIM-61, Laboratory for Medical Research 61), Department of Thoracic Surgery, University of São Paulo School of Medicine, São Paulo, Brazil; Laboratório de Investigação Médica 61 (LIM-61, Laboratory for Medical Research 61), Department of Thoracic Surgery, University of São Paulo School of Medicine, São Paulo, Brazil; Laboratory for Pleural Studies, Department of Pulmonology, University of São Paulo School of Medicine, São Paulo, Brazil; Laboratory for Pleural Studies, Department of Pulmonology, University of São Paulo School of Medicine, São Paulo, Brazil; Department of Pulmonology, University of São Paulo School of Medicine, São Paulo, Brazil

**Keywords:** Hypertension, pulmonary, Monocrotaline, Interleukin-1

## Abstract

We assessed the effects of lodenafil on hemodynamics and inflammation in the rat model of monocrotaline-induced pulmonary hypertension (PH). Thirty male Sprague-Dawley rats were randomly divided into three groups: control; monocrotaline (experimental model); and lodenafil (experimental model followed by lodenafil treatment, p.o., 5 mg/kg daily for 28 days) Mean pulmonary artery pressure (mPAP) was obtained by right heart catheterization. We investigated right ventricular hypertrophy (RVH) and IL-1 levels in lung fragments. The number of cases of RVH was significantly higher in the monocrotaline group than in the lodenafil and control groups, as were mPAP and IL-1 levels. We conclude that lodenafil can prevent monocrotaline-induced PH, RVH, and inflammation.

Pulmonary arterial hypertension (PAH) is a poor prognosis disease, which is characterized by endothelial cell proliferation, hypertrophy and proliferation of muscle cells of the media of the pulmonary arteries, reduction of the vascular lumen, and development of plexiform lesions. The reduction of the vascular lumen leads to an increase in pulmonary vascular resistance, causing right ventricle (RV) hypertrophy, cor pulmonale, and death. In addition, PAH is a public health problem, since schistosomiasis, which is one of its causes, reaches epidemic proportions in developing countries.^(^ 1 ^,^ 2 ^)^

The treatment of PAH is complex and costly, as well as requiring a multidisciplinary team. There are three major pathophysiological pathways, for which there are specific drugs available for treatment: the endothelin pathway; the nitric oxide pathway, and the prostaglandin pathway.^(^ 1 ^)^ These pathways have been discovered using experimental models of PAH, chief among which is the monocrotaline model. Many of the drugs available for the treatment of PAH have been tested using this model.^(^ 3 ^)^

The monocrotaline model is simple, inexpensive, and feasible, being routinely used in the initial analysis of drugs with potential effects on pulmonary circulation. Monocrotaline is an alkaloid derived from the seeds of the plant *Crotalaria spectabilis*; after undergoing oxidation in the liver, monocrotaline produces its toxic metabolite that will cause vasculitis and medial thickening of the pulmonary arteries and arterioles.^(^ 4 ^)^ Within 22 days after injection of monocrotaline, there is significant PAH.^(^ 5 ^)^

Lodenafil carbonate is a new phosphodiesterase-5 inhibitor consisting of two lodenafil molecules attached to a carbonate bridge that behaves as a pro-drug, releasing lodenafil as an active metabolite. Its safety in treating erectile dysfunction is well established in preclinical and clinical studies; however, it has never been tested in treating PAH.^(^ 6 ^)^

The objective of the present study was to assess the response to administration of lodenafil, in terms of hemodynamics and inflammation, in an experimental model of monocrotaline-induced PH.

All animals were handled humanely, in accordance with international standards for animal care.^(^ 7 ^)^ The study was approved by the Research Ethics Committee of the University of São Paulo School of Medicine, located in the city of São Paulo, Brazil.

Thirty male Sprague-Dawley rats (weight, 250-300 g) were randomly divided into three groups: control group, in which the animals were given a subcutaneous injection of saline (1 mL/kg) at the study outset (D0); monocrotaline group, in which the animals were given a subcutaneous injection of monocrotaline (60 mg/kg; Sigma-Aldrich, St. Louis, MO, USA) on D0; and lodenafil group, in which the animals were given a subcutaneous injection of monocrotaline (60 mg/kg; Sigma-Aldrich) on D0 and were given lodenafil p.o. (5 mg/kg) once daily between D0 and day 28 of the study (D28).

On D28, after deep sedation with xylazine hydrochloride (i.p., 0.3 mg/kg; Rompun^(r)^; Bayer, Leverkusen, Germany) and ketamine hydrochloride (i.p., 10 mg/kg; Ketalar^(r)^; Pfizer, New York, NY, USA), the animals were weighed. Subsequently, hemodynamic measurements were performed, being followed by euthanasia (abdominal aortic bleeding) and removal of heart and lung tissue.

The hemodynamic measurements were performed by inserting an umbilical catheter into the external jugular vein, the catheter being connected to a pressure transducer (HP 1295C; Hewlett-Packard, Palo Alto, CA, USA) coupled to a hemodynamic monitor (Monitox Dx 2020; Hewlett Packard), in accordance with a technique described in a previous study.^(^ 8 ^)^ Mean pulmonary artery pressure (mPAP) was thus measured.

The RV was dissected from the left ventricle (LV), the interventricular septum (S) having remained attached to the LV (LV+S). The ratio of RV weight to LV+S weight (i.e., RV/LV+S) was taken as the index of RV hypertrophy.^(^ 8 ^)^

To assess the degree of inflammation, IL-1 levels were determined with a capture ELISA using a commercial IL-1 kit (R&D System Inc., Minneapolis, MN, USA).^(^ 9 ^)^ Peptide levels were measured in frozen lung fragments.

For the statistical analysis, ANOVA with post hoc Bonferroni correction was used to compare continuous variables among the groups. Values of p < 0.05 were considered significant.

Rats in the monocrotaline group developed PAH, as shown in Figures 1 and 2, as well as experiencing a significant increase in mPAP and RV hypertrophy.

**Figure 1:**
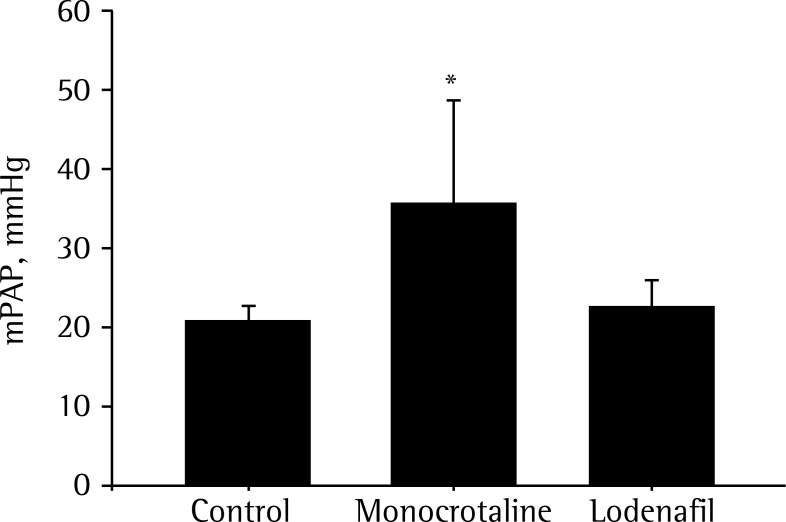
Mean pulmonary artery pressure (mPAP) in the groups studied. *p = 0.001 (monocrotaline group vs. control and lodenafil groups).

**Figure 2:**
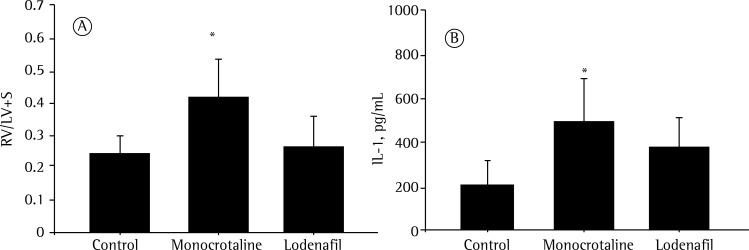
Right ventricle (RV) hypertrophy and IL-1 levels in the groups studied. In A, RV hypertrophy was determined by the ratio of RV weight to left ventricle + septum weight (LV+S). In B, IL-1 levels were determined by ELISA. *p < 0.001 (monocrotaline group vs. control and lodenafil groups for both graphs).

Rats in the lodenafil group had significantly lower mPAP than did those in the monocrotaline group, and there was no significant difference between the former and those in the control group, i.e., lodenafil prevented the development of PAH (Figure 1). The same pattern was observed for the remodeling of the RV and for IL-1 levels (Figure 2).

To our knowledge, the present study is the first to demonstrate that lodenafil was able to prevent the development of PAH in an experimental model of monocrotaline-induced disease.

It is clear that experimental PAH models do not mimic human PAH cases closely enough. There are several factors that may be related to this limitation, among which are the speed of the onset of PAH, which occurs over years in humans but progresses rapidly in animal models, and specific characteristics of the methods used to induce PAH.^(^ 5 ^)^ Specifically in the monocrotaline-induced model of disease, there is a very significant inflammatory aspect, which is not observed in the major forms of human PAH.^(^ 4 ^)^ This explains why several drugs have a marked effect in various experimental models and do not produce the same results in humans, a finding that emphasizes the care that must be taken before extrapolating results derived from experimental models directly into clinical practice.

To specifically assess the inflammatory nature of the monocrotaline model, as well as the potential anti-inflammatory properties of lodenafil, we determined IL-1 levels in lung tissue. The finding of similar IL-1 levels in lodenafil-treated compared with control rats suggests that lodenafil inhibited the inflammatory cascade characteristic of the monocrotaline model. The direct mechanism of this inhibition was not the object of our study, which is a study limitation.

Survival in monocrotaline-treated rats is significantly lower, as reported in a previous study,^(^ 8 ^)^ and this happens in parallel with progressive vascular involvement and progressive involvement of the RV. The same can be observed in humans with PAH, who have decreased survival because of the development of pulmonary vascular remodeling associated with progressive failure of the RV, with this being the leading cause of death in patients with PAH.^(^ 2 ^)^ Our study demonstrated that lodenafil prevented the increase in mPAP, as well as the remodeling of the RV, in rats with monocrotaline-induced PAH. Although we did not investigate the role of lodenafil in reversing established PAH, our findings demonstrate the therapeutic potential of lodenafil in PAH, analogously to what has been shown for other phosphodiesterase inhibitors.^(^ 3 ^)^

In conclusion, lodenafil prevented the development of PAH and the remodeling of the RV in rats subjected to an experimental model of PAH. Our findings provide the first basis for the development of clinical studies to investigate the potential of lodenafil in the treatment of human PAH.
