# Supplementary material for: Lodenafil treatment in the monocrotaline model of pulmonary hypertension in rats
Source: J Bras Pneumol. 2014 Jul-Aug;40(4):421–4. doi: 10.1590/S1806-37132014000400010 (PMC4201173; doi:10.1590/S1806-37132014000400010)
Supplement: Translation

# Translation: Tratamento com lodenafila no modelo de hipertensão pulmonar induzida por monocrotalina em ratos*

**Authors:** Igor Bastos Polonio, Milena Marques Pagliareli Acencio, Rogério Pazetti, Francine Maria de Almeida, Bárbara Soares da Silva, Karina Aparecida Bonifácio Pereira, Rogério Souza

**Affiliations:** Disciplina de Pneumologia, Faculdade de Ciências Médicas da Santa Casa de São Paulo, São Paulo (SP) Brasil; Laboratório de Pleura, Disciplina de Pneumologia, Faculdade de Medicina, Universidade de São Paulo, São Paulo (SP) Brasil; Laboratório de Investigação Médica 61 (LIM-61), Disciplina de Cirurgia Torácica, Faculdade de Medicina, Universidade de São Paulo, São Paulo (SP) Brasil; Laboratório de Investigação Médica 61 (LIM-61), Disciplina de Cirurgia Torácica, Faculdade de Medicina, Universidade de São Paulo, São Paulo (SP) Brasil; Laboratório de Pleura, Disciplina de Pneumologia, Faculdade de Medicina, Universidade de São Paulo, São Paulo (SP) Brasil; Laboratório de Pleura, Disciplina de Pneumologia, Faculdade de Medicina, Faculdade de Medicina, Universidade de São Paulo, São Paulo (SP) Brasil; Disciplina de Pneumologia, Faculdade de Medicina, Universidade de São Paulo, São Paulo (SP) Brasil

**Keywords:** Hipertensão pulmonar, Monocrotalina, Interleucina-1

## Abstract

Avaliamos os efeitos da lodenafila na hemodinâmica e inflamação no modelo experimental de hipertensão pulmonar (HP) induzida por monocrotalina em ratos. Trinta ratos Sprague-Dawley foram randomicamente distribuídos em três grupos: controle, monocrotalina (modelo experimental) e lodenafila (modelo experimental e tratado com 5 mg/kg lodenafila v.o. por 28 dias). A pressão média de artéria pulmonar (PAPm) foi obtida por cateterismo cardíaco direito. Foram determinados a hipertrofia ventricular direita (HVD) e os níveis de IL-1 em fragmentos de pulmão. O grupo monocrotalina apresentou valores significativamente maiores de PAPm, HVD e IL-1 em comparação aos grupos controle e lodenafila. Concluímos que a lodenafila pode prevenir o desenvolvimento de HP, HVD e inflamação.

A hipertensão arterial pulmonar (HAP) é uma doença de prognóstico reservado, que se caracteriza por proliferação de células endoteliais, hipertrofia e proliferação de células musculares da camada média das artérias pulmonares, redução do lúmen vascular e desenvolvimento de lesões plexiformes. A redução do lúmen vascular leva ao aumento da resistência vascular pulmonar, causando hipertrofia de ventrículo direito (VD), *cor pulmonale* e morte. Além disso, a HAP é um problema de saúde pública, já que a esquistossomose, uma de suas causas, assume proporções epidêmicas em países em desenvolvimento.^(^ 1 ^,^ 2 ^)^

O tratamento da HAP é complexo e de alto custo, assim como exige uma equipe multidisciplinar. Existem três principais vias fisiopatológicas, para as quais fármacos específicos para o tratamento estão disponíveis: a via da endotelina, a via do óxido nítrico e a via da prostaglandina.^(1) ^Essas vias foram descobertas através de modelos experimentais de HP, sendo o principal representante o modelo de monocrotalina. Através desse modelo, muitas das drogas disponíveis para o tratamento da HAP foram testadas.^(^ 3 ^)^

O modelo de monocrotalina é simples, barato e factível, sendo rotineiramente utilizado na análise inicial de fármacos com potencial ação na circulação pulmonar. A monocrotalina é um alcaloide derivado das sementes da planta *Crotalaria spectabilis,* que, após a oxidação hepática, gera seu metabólito tóxico que provocará vasculite e espessamento da camada média das artérias e arteríolas pulmonares.^(^ 4 ^)^ Após 22 dias da injeção da substância já temos uma HP importante.^(^ 5 ^)^

O carbonato de lodenafila é um novo inibidor da fosfodiesterase 5, constituído por duas moléculas de lodenafila ligadas por uma ponte de carbonato que se comporta como uma pró-droga, liberando a lodenafila como metabólito ativo. Sua segurança já está bem estabelecida para o tratamento da disfunção erétil em estudos pré-clínicos e clínicos; porém, nunca foi testada para a HAP.^(^ 6 ^)^

O objetivo do presente estudo foi avaliar a resposta à administração de lodenafila, do ponto de vista hemodinâmico e inflamatório, no modelo experimental de HAP induzida por monocrotalina.

Todos os animais receberam tratamento digno em concordância com normas internacionais de cuidados com os animais.^(^ 7 ^)^ O estudo teve a aprovação do Comitê de Ética em Pesquisa da Faculdade de Medicina da Universidade de São Paulo, na cidade de São Paulo (SP). 

Trinta ratos Sprague-Dawley machos, com peso entre 250 e 300 g, foram randomicamente distribuídos em três grupos: grupo controle - os animais receberam uma injeção subcutânea de salina (1 mL/kg) no dia de início do estudo (D0) - grupo monocrotalina - os animais receberam uma injeção subcutânea de monocrotalina (Sigma-Aldrich, Saint Louis, MO, EUA) no D0 do estudo (60 mg/kg) - e grupo lodenafila - os animais receberam uma injeção subcutânea de monocrotalina (Sigma-Aldrich) no D0 do estudo (60 mg/kg) e, entre o D0 e o dia 28 do estudo (D28), os animais receberam lodenafila v.o. (5 mg/kg) uma vez ao dia.

No D28, após sedação profunda - com cloridrato de xilazina i.p. (0,3 mg/kg; Rompun^(r)^; Bayer, Leverkusen, Alemanha) e cloridrato de cetamina i.p. (10 mg/kg); Ketalar^(r)^; Pfizer, New York, NY, EUA) - os animais foram inicialmente pesados para depois serem realizadas as medidas hemodinâmicas, seguidas do sacrifício dos animais através de sangria da aorta abdominal e, por fim, a coleta de tecidos cardíaco e pulmonar.

As medidas hemodinâmicas foram realizadas através da inserção pela veia jugular externa de um cateter umbilical conectado a um transdutor de pressão HP1295C (Hewlett-Packard, Palo Alto, CA, EUA) ligado a um monitor hemodinâmico modelo Monitox Dx 2020 (Hewlett-Packard), seguindo a técnica descrita em um estudo anterior.^(^ 8 ^)^ A pressão média da artéria pulmonar (PAPm) foi obtida através desse sistema.

O VD foi dissecado do ventrículo esquerdo (VE), o qual, por sua vez, manteve o septo interventricular (VE+S). A razão entre o peso do VD e o peso do conjunto VE+S, ou seja, VD/(VE+S), foi utilizada como um marcador de hipertrofia de VD.^(^ 8 ^)^

Para a análise da inflamação, foi determinada a concentração de IL-1 por ELISA de captura utilizando-se o kit comercial de IL-1 (R&D System Inc., Minneapolis, MN, EUA).^(^ 9 ^)^ O peptídeo foi dosado a partir de fragmentos de pulmão congelados. 

Quanto à análise estatística, as variáveis contínuas foram comparadas, entre os diferentes grupos, através de ANOVA e análise *post hoc* com correção de Bonferroni. Foram considerados significativos os valores de p < 0,05.

O grupo monocrotalina desenvolveu HAP, como mostram as Figuras 1 e 2, com aumento significativo da PAPm e hipertrofia de VD.

**Figura 1:** Pressão média da artéria pulmonar (PAPm) nos grupos estudados. *p = 0,001 (grupo monocrotalina vs. grupos controle e lodenafila).

**Figura 2:** Hipertrofia de ventrículo direito (VD) e níveis de IL-1 nos grupos estudados. Em A, a hipertrofia do VD foi determinada pela razão entre o peso do VD e o peso do ventrículo esquerdo + septo (VE+S). Em B, níveis de IL-1 por ELISA. *p < 0,001 (grupo monocrotalina vs. grupos controle e lodenafila para ambos os gráficos).

O grupo lodenafila apresentou PAPm significativamente menor do que o grupo monocrotalina, sem diferença significativa quando comparado ao grupo controle, ou seja, a lodenafila preveniu o desenvolvimento de HAP (Figura 1). O mesmo padrão foi observado em relação ao remodelamento do VD e aos níveis de IL-1 (Figura 2).

Até onde sabemos, o presente estudo é o primeiro a demonstrar que a lodenafila foi capaz de prevenir o desenvolvimento de HAP no modelo experimental induzido com monocrotalina.

É evidente que os modelos experimentais em HAP não mimetizam adequadamente a doença que ocorre em seres humanos. Vários são os motivos que podem estar relacionados a essa limitação, desde o tempo necessário para instalação da HAP, ao longo de anos nos seres humanos e de forma rapidamente progressiva nos modelos animais, assim como características específicas dos métodos utilizados para indução da HAP.^(^ 5 ^)^ Especificamente no modelo induzido por monocrotalina, há um aspecto inflamatório muito significativo, não observado nas principais formas de HAP em humanos.^(^ 4 ^)^ Isso explica o porquê de vários fármacos terem um efeito marcante em diversos modelos experimentais e não apresentarem os mesmos resultados em seres humanos, enfatizando o cuidado que deve ser tomado antes de se extrapolar resultados oriundos de modelos experimentais diretamente para a prática clínica.

Especificamente para avaliar o caráter inflamatório do modelo de monocrotalina, assim como as potenciais propriedades anti-inflamatórias da lodenafila, foram determinados os níveis de IL-1 no tecido pulmonar. O achado de níveis de IL-1 semelhantes aos do grupo controle nos ratos tratados com lodenafila sugere que a lodenafila tenha inibido a cascata inflamatória característica do modelo de monocrotalina. O mecanismo direto dessa inibição não foi objeto de nosso estudo, caracterizando uma limitação do mesmo.

A sobrevida de ratos tratados com monocrotalina é significativamente menor, conforme um estudo publicado anteriormente,^(^ 8 ^)^ de forma paralela ao progressivo acometimento vascular e do VD. O mesmo pode ser observado em relação a seres humanos com HAP, que apresentam sobrevida diminuída devido ao desenvolvimento de remodelamento vascular pulmonar associado à falência progressiva do VD, sendo essa a principal causa de morte em pacientes com HAP.^(^ 2 ^)^ Nosso estudo demonstrou que a lodenafila preveniu a elevação da PAPm, assim como o remodelamento do VD nos ratos com HAP em decorrência da monocrotalina. Embora não tenhamos avaliado o papel da lodenafila na reversão da HAP já estabelecida, nosso estudo evidencia o potencial terapêutico da lodenafila na HAP, de forma análoga ao já demonstrado para outros inibidores de fosfodiesterase.^(^ 3 ^)^

Em conclusão, a lodenafila preveniu o desenvolvimento de HAP e o remodelamento do VD em ratos submetidos a um modelo experimental de HAP. Nossos achados fornecem a primeira base para o desenvolvimento de estudos clínicos avaliando o potencial da lodenafila no tratamento da HAP em seres humanos.
